# Complete mitochondrial genome and phylogenetic analysis of Huangshan Black chicken (*Gallus gallus*)

**DOI:** 10.1080/23802359.2020.1860694

**Published:** 2021-01-27

**Authors:** Sihua Jin, He Zang, Peili He, Tingting Jiang, Shenqiang Pan, Zhaoyu Geng

**Affiliations:** College of Animal Science and Technology, Anhui Agricultural University, Hefei, P.R. China

**Keywords:** Huangshan Black chicken, mitochondrial genome, phylogenetic analysis

## Abstract

In this study, we sequenced the complete mitochondrial genome (mitogenome) of the Huangshan Black chicken (HBC). Results showed that the complete HBC mitogenome was 16,785 bp in size, comprising 22 transfer RNA genes, 2 ribosomal RNA genes, 13 protein-coding genes, and 1 non-coding control region (D-loop). The overall nucleotide composition was 32.5% for C, 30.3% for A, 23.7% for T, and 13.5% for G. Phylogenetic analysis showed that the HBC mitogenome was clustered with Xianju chicken, which belonged to the haplogroup D2. Our results therefore demonstrate that the origin of HBC corresponds to haplogroup D2 distribution and might have at least one maternal lineage originated from Southeast Asia.

The domestic chicken is an important source of protein for humans, providing large quantities of meat and eggs. The Huangshan Black chicken (HBC, *Gallus gallus*) is one of the most important domestic chicken breeds in Anhui province, which was documented as a local poultry genetic resource in 2009. It is very well-known for its strong disease resistance, adaptability, superior meat quality, and high polyunsaturated fatty acids percentage (Yang et al. 2018). However, the genetic resources of HBC should be preserved because increased use of highly productive commercial breeds has reduced genetic diversity in chickens (Gao et al. 2017). In this study, we sequenced the complete mitochondrial genome (mitogenome) of HBC for the first time and used these data to perform phylogenetic analyses.

Blood samples of HBC were collected from Yi County (E117°38′–118°6′, N29°47′–30°11′), Huangshan, China. Genomic DNA was isolated from the specimens (voucher no. HBC190916) and stored at −70 °C in the laboratory of Poultry Genetics and Breeding, College of Animal Science and Technology, Anhui Agricultural University. DNA integrity was evaluated using 1.0% agarose gel electrophoresis. We then performed whole-genome shotgun sequencing using paired-end Illumina NovaSeq, with a read length of 150 bp (TSINGKE Biological Technology, Beijing, China). The genome was assembled by SPAdes (Bankevich et al. 2012), annotated by GeSeq (Tillich et al. 2017), and tRNA Scan-SE 1.21 (http://lowelab.ucsc.edu/tRNA Scan-SE/) was used to search for tRNA genes in the genomic sequences.

The complete mitogenome was 16,785 bp in size and typical in structure, containing 13 protein-coding genes, 22 transfer RNA genes, 2 ribosomal RNA genes, and one non-coding control region (D-loop) (GenBank accession No. MT762346). Base composition was 32.5% for C, 30.3% for A, 23.7% for T, and 13.5% for G. The start codon for all protein-coding genes was ATG, except *COX1*, which had GTG as the start codon. These genes had 15 spaces and 7 overlaps, both 1–9 bp in length. Eight tRNA genes (*tRNA^Gln^, tRNA^Ala^, tRNA^Asn^, tRNA^Cys^, tRNA^Tyr^, tRNA^Ser^, tRNA^Pro^*, and *tRNA^Glu^*) and one protein-coding gene (*ND6*) were encoded on the L strand; the rest of the genes were encoded on the H strand. We identified three different complete stop codons: TAG for *ND2*, AGG for *COX1*, and TAA for *ND1*, *COX2*, *ATPase8*, *ATPase6*, *ND3*, *ND4L*, *ND5*, *Cytb*, and *ND6*. For *COX3* and *ND4*, the stop codon was incomplete (T––) and was located in the 5′ terminal of the adjacent gene (Anderson et al. 1981). The D-loop region was 1232 bp in length and was located between *tRNA^Glu^* and *tRNA^Phe^*.

A maximum-likelihood (ML) tree was constructed using MEGA 7.0 (Kimura 1980; Kumar et al. 2016) with 1000 bootstrap replicates, based on the complete HBC mitogenome sequence and 14 chicken breeds sequences from the National Center for Biotechnology Information (NCBI). Phylogenetic analysis (Figure 1) showed that the HBC mitogenome was clustered with Xianju chicken. Previous studies have shown that the mitogenome of Xianju chicken (GU261677) belonged to haplogroup D2, which most likely originated from Southeast Asia (Liu et al. 2006; Miao et al. 2013). We therefore conclude that the origin of HBC corresponds to haplogroup D2 distribution and might have at least one maternal lineage originated from Southeast Asia. This study will provide fundamental data for conservation, breeding, and further genetic studies of HBC and other indigenous chicken breeds.

**Figure 1.**
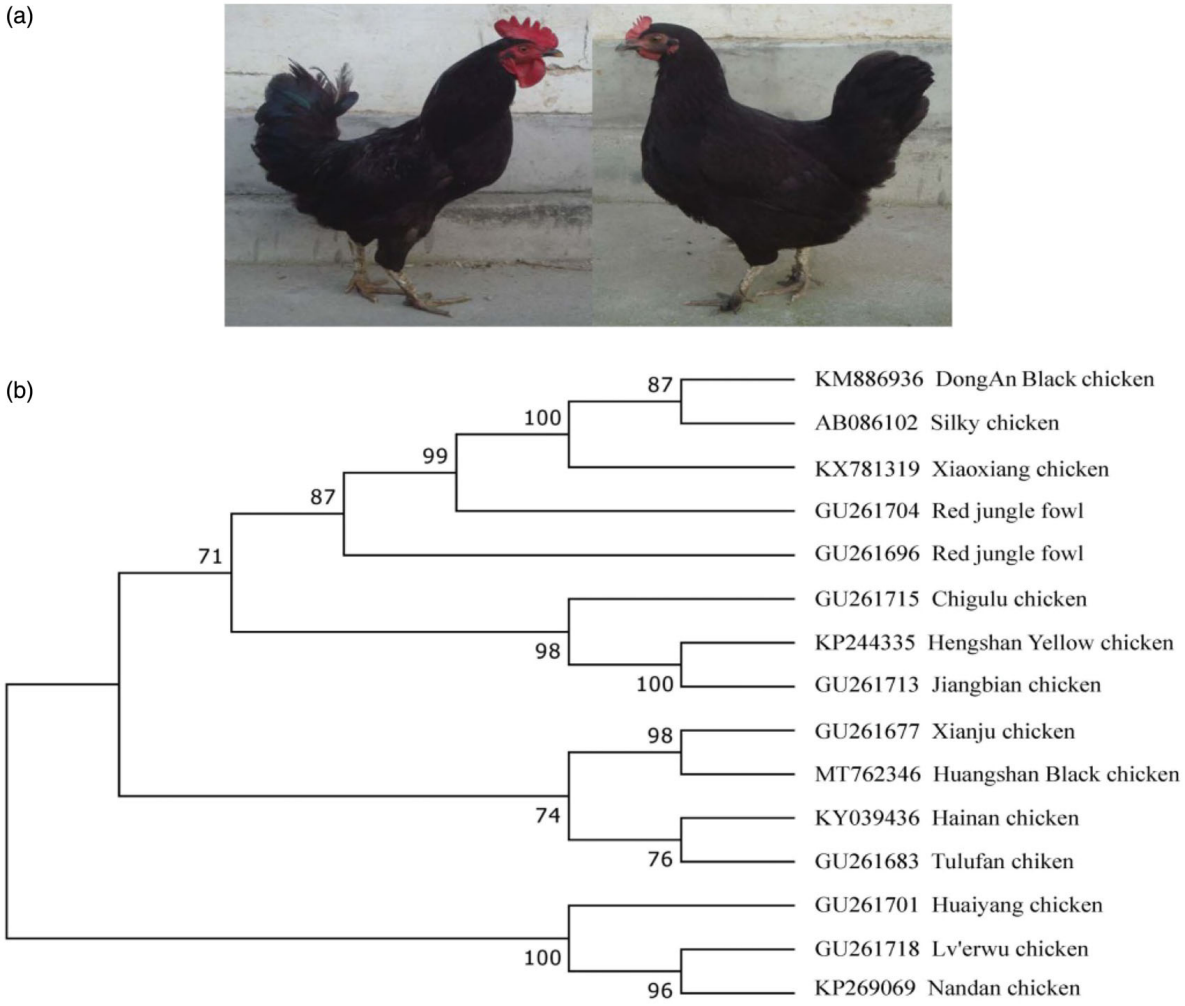
Characteristic of Huangshan Black chicken (HBC) and phylogenetic analysis based on the complete mitochondrial genome sequences of HBC. (a) Phenotypes of HBC. (b) The Phylogenetic tree. A maximum-likelihood (ML) tree was constructed from the complete HBC mitogenome and 14 chicken breed sequences. The 14 sequences were obtained from GenBank (accession numbers on the figure).

## Data Availability

The genome sequence data that support the findings of this study are openly available in GenBank of NCBI at (https://www.ncbi.nlm.nih.gov/) under the accession no. MT762346. The associated BioProject, SRA, and BioSample numbers are PRJNA678580, SRP292644, and SAMN16807199, respectively.
